# Fe^II^_4_L_4_ tetrahedron binds and aggregates DNA G-quadruplexes†

**DOI:** 10.1039/d1sc04430c

**Published:** 2021-10-07

**Authors:** Jinbo Zhu, Zhiqiang Yan, Filip Bošković, Cally J. E. Haynes, Marion Kieffer, Jake L. Greenfield, Jin Wang, Jonathan R. Nitschke, Ulrich F. Keyser

**Affiliations:** Cavendish Laboratory, University of Cambridge JJ Thompson Avenue Cambridge CB3 0HE UK ufk20@cam.ac.uk; Yusuf Hamied Department of Chemistry, University of Cambridge Lensfield Road Cambridge CB2 1EW UK jrn34@cam.ac.uk; State Key Laboratory of Electroanalytical Chemistry, Changchun Institute of Applied Chemistry, Chinese Academy of Sciences Changchun Jilin 130022 P. R. China; Department of Chemistry and of Physics, State University of New York at Stony Brook Stony Brook New York 11794-3400 USA

## Abstract

Since the discovery of the G-quadruplex (G4) structure in telomeres in 1980s, studies have established the role it plays in various biological processes. Here we report binding between DNA G4 and a self-assembled tetrahedral metal-organic cage **1** and consequent formation of aggregates, whereby the cage protects the DNA G4 from cleavage by S1 nuclease. We monitor DNA–cage interaction using fluorescence spectroscopy, firstly by quenching of a fluorescent label appended to the 5′ end of G4. Secondly, we detect the decrease in fluorescence of the G4-selective dyes thioflavin-T and Zn-PPIX bound to various DNA G4 sequences following the addition of cage **1**. Our results demonstrate that **1** interacts with a wide range of G4s. Moreover, gel electrophoresis, circular dichroism and dynamic light scattering measurements establish the binding of **1** to G4 and indicate the formation of aggregate structures. Finally, we find that DNA G4 contained in an aggregate of cage **1** is protected from cleavage by S1 nuclease.

## Abstract 2

We find Fe^II^_4_L_4_ binds to G-quadruplex and forms aggregates. G-quadruplex in the aggregates is protected from digestion by S_1_ nuclease.

## Graphical abstract

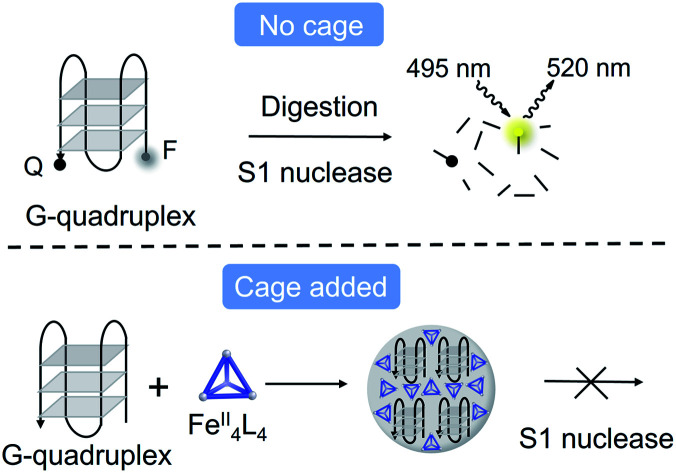

## Introduction

The DNA G-quadruplex (G4) is a four-stranded helical structure formed by the stacking of two or more planar G-tetrads on top of each other, with every G-tetrad containing four guanine bases associated through Hoogsteen hydrogen bonds.^1^ G4-forming sequences were first discovered in telomeres and have since become potential anticancer drug targets.^2–4^ G4-interactive molecules can be used to inhibit telomerase activity and induce telomeric dysfunction.^5–7^ Furthermore, DNA G4s are present in the non-telomeric genome, *e.g.* promoter regions,^8,9^ and are involved in cellular events such as replication,^10^ transcription,^11^ translation^12^ and DNA damage.^13^ The G4 structure is also common in many G-rich DNA aptamers.^14,15^ Owing to their significant role in biology and biosensing, G4 structures have attracted great interest recently. Much of this interest has focused upon the design and study of G4 ligands. Many compounds able to bind DNA G4 have been reported, such as small molecules and metal complexes, some of which are promising candidates as anticancer drugs or biological process regulators.^16–20^

Self-assembled metal–organic cages are useful for a range of areas including catalysis, biomedicine, molecular sensing, gas absorption and molecular separation.^21–25^ Previous studies have shown that various metal–organic assemblies can recognize the structure of DNA G4 and alter its conformation.^5–7,26–30^ The structural tunability and encapsulation ability of coordination cages render them applicable for biosensing, drug delivery and cancer therapy.^31–34^ However, only a few metal–organic cages for biomedical applications have been reported so far.^35^

Two factors may have limited the widespread biomedical uses of metal–organic cages. First, the insoluble aromatic ligands and the dynamic nature of the interactions involved in the cage assembly cause many cages to be poorly soluble and stable in water.^36^ Second, the detection of intermolecular binding between cages and biomolecules can be challenging, compared to the well-established procedures in place for fluorescent or colorimetric probes.^37,38^

**Fig. 1:**
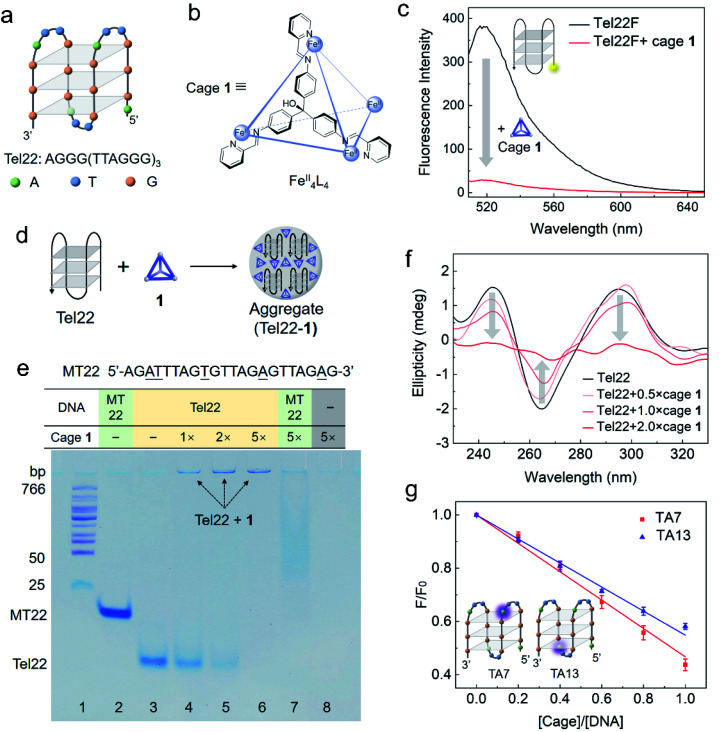
Interaction between DNA G4 (Tel22) and cage **1** studied by fluorescence spectroscopy, polyacrylamide gel electrophoresis (PAGE), circular dichroism (CD) spectroscopy and 2-aminopurine label. (a) Structure of the basket-type G4 in TSN buffer solution (10 mM Tris–H_2_SO_4_, 10 mM Na_2_SO_4_, pH 7.5) formed by Tel22. (b) Schematic structure of Fe^II^_4_L_4_ cage **1**, showing the chemical structure of one face-capping L ligand (ESI Section S1.2†). (c) Fluorescence spectra (ex. 495 nm) of Tel22F (100 nM) without and with cage **1** (200 nM) in TSN buffer solution (10 mM Tris–H_2_SO_4_, 10 mM Na_2_SO_4_, pH 7.5). (d) Cartoon showing the formation of aggregates between Tel22 and **1**. (e) Photograph of a polyacrylamide gel comparing Tel22 (20 μM) and a single strand MT22 (20 μM) with and without cage **1** in 0.5 × TB buffer with Na^+^ (44.5 mM Tris, 44.5 mM boric acid, 10 mM Na_2_SO_4_, pH 8.0). A DNA ladder was added in lane 1, MT22 was added in lane 2 and 7, and Tel22 was added in lanes 3–6. Different concentrations of **1** were premixed with DNA as indicated (equivalents relative to DNA). The gel was stained using stains-all instead of fluorescent dye to avoid cage quenching. (f) CD spectra of Tel22 (10 μM) in TSN buffer with different concentrations of **1** (measured at 298 K). (g) Fluorescence titration of 5 μM TA7 or TA13 by **1** in TSN buffer based on a 2-aminopurine fluorescence assay to locate the preferred binding site. The 7^th^ and 13^th^ adenines at the two ends of G4 are substituted by 2-aminopurine in TA7 and TA13, respectively. *F*_0_ is the fluorescence intensity of DNA without **1**. The error bars are based on three repetitions for each data point.

Recently, we reported that the water-soluble Fe^II^_4_L_4_ tetrahedral cage **1** (Fig. 1b)^39^ can bind to unpaired nucleotides in DNA and thereby quench a proximate fluorescent label.^33,40^ The presence of unpaired nucleotides in G4 inspired us to investigate whether cage **1** can interact with G4. Given the biological significance of telomeres for cell aging and death,^2–4^ human telomeric DNA G4 dAG_3_[T_2_AG_3_]_3_ (Tel22, Table S1†) was used as a model G4 in this work. This DNA strand folds into a basket conformation in the presence of Na^+^ (Fig. 1a) or into a hybrid structure (three parallel G strands and one antiparallel G strand) in the presence of K^+^ (Fig. S1a†).^41^ Cage **1** was found to interact with Tel22, as evidenced by the quenching of the fluorescent dyes label or bound on the G4 strand. However, in contrast to binding to single-stranded DNA (ssDNA), DNA G4 formed aggregates with **1**, as observed by gel electrophoresis, a decrease of circular diagram (CD) signal, and resistance to S1 nuclease cleavage. Our findings lead to a better understanding of the interaction between metal–organic cages and DNA, and to new potential applications.

## Results and discussion

### Cage **1**-mediated quenching of fluorescent label on G4

Cage **1** is known to quench the fluorescent dye FAM (fluorescein) when it was labelled on DNA but it does not affect the fluorescence of free FAM in solution.^33^ The selective quenching of fluorescence allow us to gauge the strength of the cage–G4 interaction with bulk techniques. As shown in Fig. 1c, the cage quenched the fluorescence of Tel22F (FAM labelled dAG_3_[T_2_AG_3_]_3_, ESI Table S1†) in a solution containing Na^+^. This observation indicates that **1** interacts with the basket-type structure of G4, formed by the Tel22 sequence. The fluorescence response as a function of cage concentration is given in Fig. S2.† The binding was also confirmed by molecular docking carried out at the individual molecular level for the basket structure of Tel22 in Na^+^ solution (ESI Section S1.8 and Fig. S13†). The results indicate that the cage may prefer side-binding onto the basket G4 structure with an affinity of −11.00 kcal mol^−1^.

Similar fluorescence results were obtained for the hybrid-type G4 structure in a solution containing K^+^ (Fig. S1b†). Furthermore, the cage shows a stronger quenching ability and higher affinity towards G4 than double-stranded DNA (Fig. S3 and Table S2†). Thus, the complementary strand cTel22 can be used to tune the interaction between Tel22 and cage **1** (Fig. S4†),^42^ which indicates that the fluorescence quenching is reversible due to the cage interacting directly with the DNA nucleotides rather than the labelled dye.

### Interaction between G4 and cage **1** causes aggregation

Native polyacrylamide gel electrophoresis (PAGE) was utilized to study the intermolecular interactions. Strand MT22 (ESI Table S1†), generated from substituting several bases of Tel22 (underlined in Fig. 1e), is used as a non-G4-forming mutant control in the gel.^43^ FAM-labelled MT22 also interacts with cage **1** as a ssDNA, and shows a similar quenching curve to Tel22F (Fig. S5 and Table S2†). Since cage **1** quenches nearby fluorophores,^33^ we employed the metachromatic dye stains-all instead of a fluorescent dye to stain the gel and visualize the DNA bands.

Tel22 folds into a basket-type G4 structure in the Na^+^-containing electrophoresis buffer, whereas MT22 does not fold. As a result of its compact conformation, Tel22 shows a greater PAGE mobility than the single-stranded MT22 (lanes 2 and 3 in Fig. 1e). Upon increasing the amount of cage **1** (lanes 4 to 6), the bands corresponding to folded Tel22 are observed to weaken, while new bands at the top darken. We attribute the weakening to interactions with **1**, and the new band to consist of an adduct between G4 and cage **1**.

Binding between **1** and G4 is inferred to be due to positively charged **1** serving as a counterion to the negatively charged DNA. The low mobility on the gel is a consequence of the formation of aggregates, as illustrated in Fig. 1d. These aggregates reduce the mobility of the complex. Without DNA, positively charged **1** was driven into solution by the electrophoresis voltage, resulting in a blank lane (lane 8). The band of MT22 in the presence of **1** (5 equiv.) in lane 7 is observed to shift and smear, which is inferred to be due to the binding of **1** to the single-stranded MT22. However, no new band was found at the top of lane 7, in contrast with lane 6, which indicates that **1** formed aggregates with G4. The higher hydrophobicity of the stacked G-tetrad-containing folded G4 structure, as compared to ssDNA, accounts for this different behaviour.^44,45^

As a useful technique for characterization of G4 structures, CD was employed to investigate the change of DNA G4 upon the addition of cage **1**.^46^ In the absence of cage **1**, the characteristic CD bands at 295, 240, and 265 nm are consistent with G4 adopting an antiparallel basket-type structure (Fig. 1f). The intensities of these CD bands decrease upon addition of cage **1** (no CD signal was observed for cage **1** itself), indicating interaction between the cage and G4, as formulated from the experimental results shown in Fig. 1c and e. Analogous CD results were also obtained in solutions containing K^+^ for the hybrid quadruplex (Fig. S6†). The characteristic CD signals of G4 almost completely disappeared in the presence of a two-fold excess of the cage, which we infer to be due to the condensation of Tel22 DNA in the aggregates as we found in the gel (Fig. 1e) and precipitation of the larger aggregates.^47^

In order to better understand the interaction between **1** and G4, 2-aminopurine (2-Ap), a naturally fluorescent analogue of adenine, was substituted for the specific adenines in the DNA sequence.^48,49^ As noted in ESI Table S1,† the 7^th^ or 13^th^ A of Tel22 was replaced by 2-Ap to yield the two fluorescent analogues TA7 and TA13, respectively. The fluorescence intensities of both analogues decreased upon increasing the cage concentration (Fig. 1g). In a folded structure, the two adenines are present at the two ends of the G4 structure, which has been observed to lead to a difference in quenching efficiency.^7,48^ However, similar quenching effects were observed here by adding cage **1** to each of these two 2-Ap-containing strands. We infer that this similar quenching behaviour resulted from the side-binding mode (Fig. S13†) and the formation of aggregates after the addition of **1**, allowing both sites to quench to a similar degree in the crowded internal environments of the aggregates.

### Verification of aggregates

**Fig. 2:**
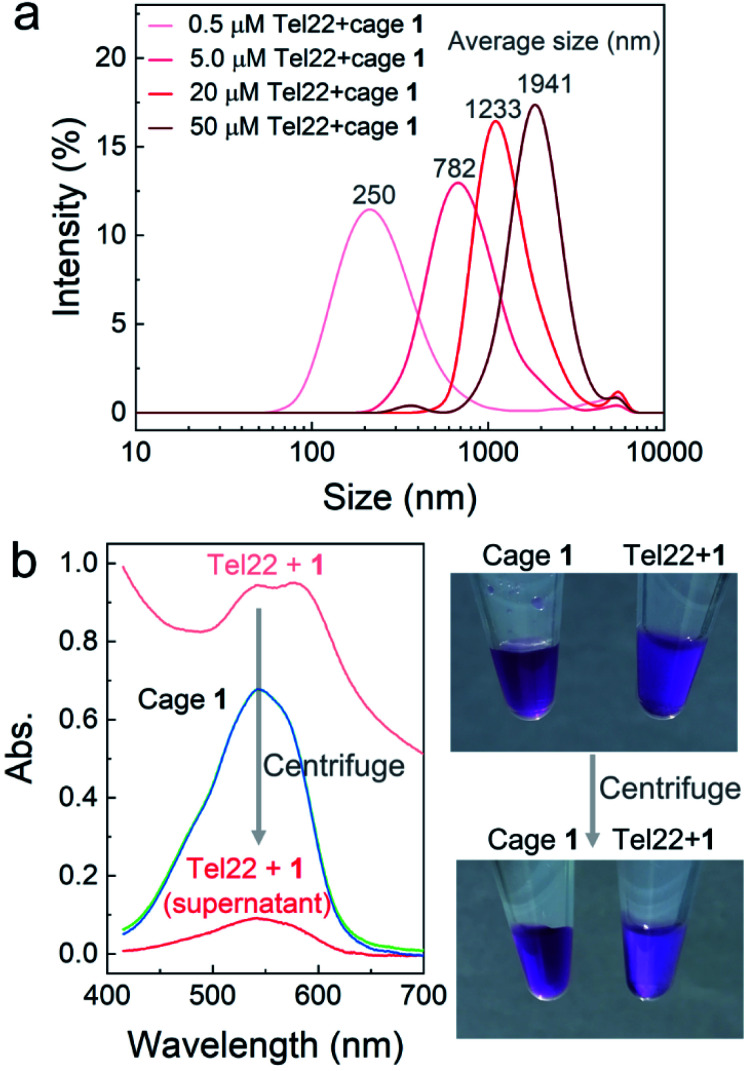
Mixture of Tel22 and **1** investigated by dynamic light scattering (DLS) and UV-vis spectroscopy. (a) DLS intensity distributions of the Tel22–**1** mixtures at different concentrations. The molar ratio of Tel22 to **1** was kept at 1 : 2 in all mixtures. (b) Absorption spectra of the Tel22–**1** mixture before (light red) and after (dark red) centrifugation; 100 μM Tel22 and 200 μM cage **1** were mixed and centrifuged as shown in the photos at right. The mixture and supernatant were both diluted 10 times for the absorbance measurement. Cage **1** alone at the same concentration was centrifuged simultaneously for comparison. The spectra of **1** before and after centrifugation are shown together as green and blue curves, respectively.

To further confirm aggregation and measure the size of the aggregates, dynamic light scattering (DLS) was applied to investigate the mixtures of Tel22–**1** (molar ratio 1 : 2) at different concentrations. As shown in Fig. 2a, the average diameter of the aggregates increased with the concentration of Tel22 and **1**. However, DLS results with uniform intensity distribution were not obtained from the MT22-**1** and dsDNA-**1** mixtures (Fig. S7†), which indicates a discrete aggregation mechanism of the G4–**1** complex, in contrast with poorly-structured aggregates for non-G4 DNA. When aggregates were at the micron scale, at 50 μM concentrations of Tel22 and **1**, they were directly observed using an optical microscope (Fig. S8†). The formation of aggregates also reduced the transmissivity and led to a high baseline in the absorption spectra (light red curve, Fig. 2b). After centrifugation, the supernatant clarified to low absorbance (dark red curve, Fig. 2b), with the aggregates precipitating and concentrating on the wall of the tube, as shown in the photo of Fig. 2b. Cage **1** alone at the same concentration was unaffected by centrifugation (overlapped green and blue curves, Fig. 2b), indicating that Tel22 interacted with **1** and precipitated at higher concentrations.

### Interaction between various G4 sequences and cage **1**

**Fig. 3:**
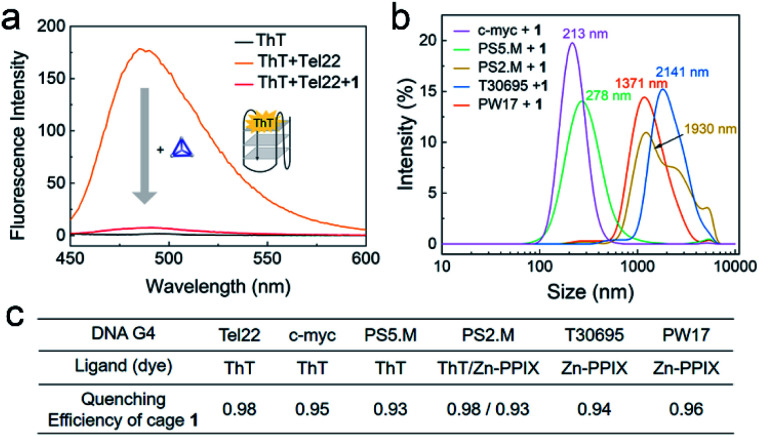
(a) Fluorescence spectra of ThT (1 μM, black) and Tel22 (1 μM) without (orange) or with (red) cage **1** (2 μM) excited at 425 nm in TSK buffer (10 mM Tris–H_2_SO_4_, 10 mM K_2_SO_4_, pH 7.5). (b) DLS results from the mixtures of **1** and various G4s in TSK buffer. The concentrations of **1** and DNA G4 were 10 μM and 5 μM, respectively. Average sizes of the aggregates are given above the spectra. (c) Table of the QE of cage **1** (2 μM) for different DNA G4 sequences (1 μM, ESI Table S1†) in the presence of these dyes. Spectra are presented in Fig. S9.†

The interaction between different G4 sequences and cage **1** were studied in an accessible and economical way by employing G4-sensitive dyes. These fluorescent dyes, such as thioflavin T (ThT), protoporphyrin IX (PPIX), Zn-protoporphyrin IX (Zn-PPIX) and *N*-methyl mesoporphyrin IX (NMM), are an important class of probes for G4 sensing, as their fluorescence intensity is enhanced upon binding with G4.^50–52^ We found that **1** reduces the fluorescence of a mixture of ThT and Tel22 by more than a factor of 10 (Fig. 3a). The enhanced fluorescence of ThT or Zn-PPIX with a series of different G4 sequences was also found to significantly decrease in the presence of **1** (Fig. 3c and S9†), resulting in similar quenching efficiencies. These results indicated that **1** interacted with various G4 sequences.

The above observations led us to infer that the interaction between **1** and G4s resulted in the formation of aggregates, and that the fluorescence of the G4-sensitive dyes were quenched by the cage. The DLS results of the mixture of **1** and a series of G4s in Fig. 3b supported this hypothesis. Additionally, the G4 sequence and conformation clearly influence the size of aggregates, which will be further studied in the future.

### Cage **1** inhibits S1 nuclease digestion

Next, we probed the ability of **1** to regulate the activity of the S1 single-stranded endonuclease enzyme, which captures and hydrolyses DNA G4 into segments and 5′-mononucleotides,^53^ envisioning that cage **1** might compete with S1 to bind to the G4 Tel22.

**Fig. 4:**
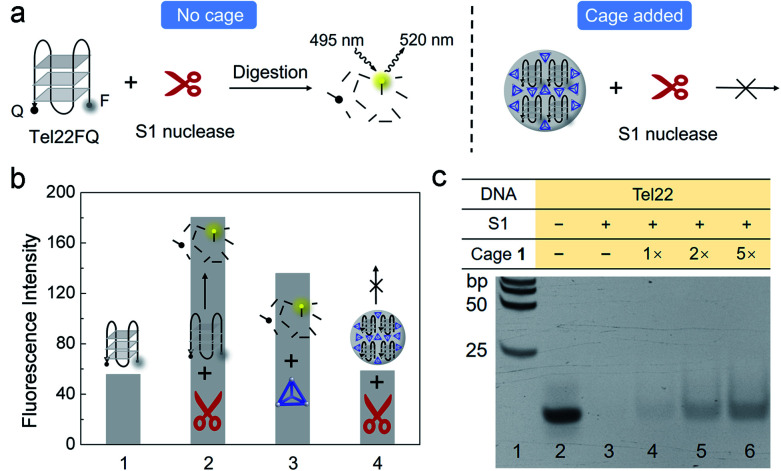
Fluorescence and gel electrophoresis of the digestion of G4 by S1 in the presence and absence of cage **1**. (a) Scheme for the S1 nuclease cleavage of Tel22FQ with and without **1**. (b) Fluorescence intensity of (1) Tel22FQ before digestion; (2) Tel22FQ after digestion by S1; (3) Tel22FQ after digestion by S1 in the presence of **1** (added afterwards); (4) Tel22FQ in the presence of **1** added before S1 digestion. The concentrations of Tel22FQ and **1** were 4 μM and 20 μM, respectively, for the S1 digestion, and the solutions were diluted 40 times for fluorescence measurement. (c) S1 digestion of Tel22 analyzed by 15% PAGE in 0.5 × TB buffer (44.5 mM Tris, 44.5 mM boric acid, pH 8.0). All samples were preheated to disassemble the cage before gel electrophoresis. The contents of the mixture loaded into each lane is indicated in the table above the gel. A DNA ladder was added into the first lane.

FAM and a quencher were attached to the 5′ and 3′ end of Tel22 (Tel22FQ), respectively (Fig. 4a), so that the fluorescence was quenched when Tel22FQ was folded into its G4 structure (bar 1 in Fig. 4b). The fluorescence was recovered, however, after S1 digestion (bar 2). When Tel22FQ was premixed with **1** to form aggregates before digestion, the fluorescence remained quenched (bar 4), thus indicating that **1** protected the G4 from the action of S1. As the gel, CD, DLS and absorption results shown in Fig. 1 and 2, the aggregates may precipitate out of solution, rendering the majority of the G4 inaccessible to S1. When **1** was added after G4 was exposed to S1 (bar 3), fluorescence was observed. This observation indicated that the G4 was digested by S1 in the absence of protective **1**. Furthermore, cage **1** did not affect the fluorescence of the digested segments.

Cage regulation of S1 digestion of Tel22 was further evidenced by gel electrophoresis (Fig. 4c). No band was observed in lane 3, indicating that nuclease S1 efficiently cleaved Tel22. Upon increasing the concentration of cage (lanes 4 to 6) added before S1 digestion, the band corresponding to intact Tel22 increased in intensity, despite the presence of nuclease S1. In contrast with the gel analysis shown in Fig. 1e, the Fig. 4c samples were heated to 95 °C for 3 min in the presence of EDTA (22.8 mM), conditions which were observed to result in the disassembly of **1**, prior to gel electrophoresis (Fig. S10 and S11†). This treatment resulted in release of the G4 strand from the aggregates, enabling it to migrate into the gel in Fig. 4c. These observations suggested that larger amounts of **1** led to a greater degree of protection. By contrast, cage **1** did not protect MT22 from S1 cleavage under the same conditions (Fig. S12†), which is consistent with the gel result in Fig. 1e.

## Conclusions

Fe^II^_4_L_4_ cage **1** was thus observed to interact with G4 structures and form aggregates. This intermolecular interaction between the cage and a labelled G4 strand Tel22 was detected using fluorescence spectroscopy, due to the fluorescence quenching property of the cage. Unlike other common G4 ligands which bind to G4 structures, cage **1** is found to form aggregates with quadruplexes, as evidenced by CD, PAGE, DLS and UV-vis absorption analyses. Moreover, the aggregation of cage **1** and Tel22 prevents digestion by the S1 nuclease. This study paves the way for the development of new cages, with different ligands and metal ions, of greater thermal stability, binding selectivity, biocompatibility and being capable of being loaded with small-molecule guests that interact with DNA. We foresee more G4 related bioprocesses being tuned by metal–organic cages, leading to biological applications such as gene expression regulation or drug delivery.

## Data availability

Data for this work are available at https://doi.org/10.5281/zenodo.5557929.

## Author contributions

JZ, UFK and JRN conceived the project; JZ, ZY and FB designed the experiments; JZ performed the majority of the experiments, analysed the data and wrote the manuscript; CJEH and MK synthesized the cage Fe^II^_4_L_4_; FB and JLG contributed to the CD spectra measurement; ZY and JW contributed to the molecular docking. All authors discussed the results and commented on the manuscript.

## Conflicts of interest

There are no conflicts to declare.

## Supplementary Material

SC-012-D1SC04430C-s001
