# Supplementary material for: FeII4L4 tetrahedron binds and aggregates DNA G-quadruplexes
Source: Chem Sci. 2021 Oct 7;12(43):14564–9. doi: 10.1039/d1sc04430c (PMC8580047; doi:10.1039/d1sc04430c)
Supplement: SC-012-D1SC04430C-s001 [file SC-012-D1SC04430C-s001.pdf]

Electronic Supplementary Material (ESI) for Chemical Science.
This journal is © The Royal Society of Chemistry 2021

Supplementary data for

$Fe^{II}_4L_4$ tetrahedron binds and aggregates DNA G-quadruplexes

Jinbo Zhu,[a] Zhiqiang Yan,[c] Filip Bošković,[a] Cally J. E. Haynes,[b] Marion Kieffer,[b] Jake L. Greenfield,[b] Jin Wang,[d] Jonathan R. Nitschke,*[b] and Ulrich F. Keyser,*[a]

[a] Cavendish Laboratory, University of Cambridge, JJ Thomson Avenue, Cambridge, CB3 0HE, United Kingdom; [b] Yusuf Hamied Department of Chemistry, University of Cambridge, Lensfield Road, Cambridge, CB2 1EW, United Kingdom; [c] State Key Laboratory of Electroanalytical Chemistry, Changchun Institute of Applied Chemistry, Chinese Academy of Sciences, Changchun 130022, P. R. China; [d] Department of Chemistry and Department of Physics, State University of New York at Stony Brook, Stony Brook, New York 11794-3400, United States.

S1 Experimental details

S1.1 Materials

DNA oligonucleotides were purchased from Integrated DNA Technologies, Inc. (IDT). S1 nuclease was purchased from Thermal Scientific. Other chemicals were of reagent grade and were used without further purification. The sequences of DNA strands are listed in Table S1.

S1.2 Synthesis of cage

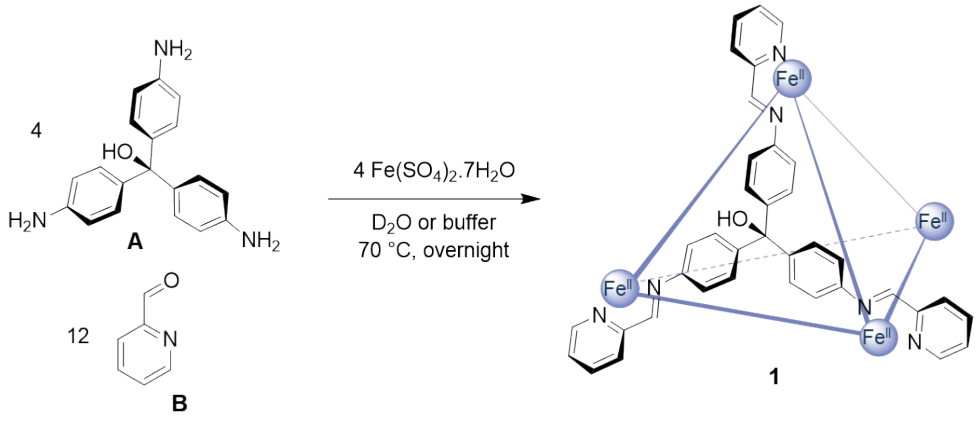

Scheme S1. Subcomponent self-assembly of cage **1** in aqueous solution.

The basic synthesis process was shown in Scheme S1. Stock solutions of cage **1** were prepared at a concentration of 10 mM and diluted as required. Detailed synthesis protocol, NMR spectrum and stability study of cage **1** can be found in the previous work.[1]

S1.3 Fluorescence spectroscopy measurement and analysis

Tel22F was heated at 88°C for 5 min in TSN buffer (10 mM Tris-H_2SO_4, 10 mM Na_2SO_4, pH 7.5) and then slowly cooled down to room temperature (20 °C). Cage **1** diluted to the desired concentration in TSN buffer was added into the DNA solution. Final concentrations of cage **1** and DNA were 0.2 μM and 0.1 μM for fluorescence measurement respectively, if not indicated otherwise. Cage and DNA were quickly mixed just before the measurement. The fluorescence emission spectra were collected from 510 nm to 650 nm and kinetic data were recorded at 520 nm with an excitation of 495 nm in a 10 mm path-length quartz cuvette using a Cary Eclipse fluorescence spectrophotometer (Agilent Technologies, U.S.A.).

In the test of fluorescent base 2-Aminopurine, TA7 or TA13 was annealed in TSN buffer as above and mixed with various concentrations of cage **1**. It was excited at 305 nm and the emission intensity was collected at 370 nm.

In the label-free dye measurement, 1 μM G-quadruplex DNA was annealed in TSK buffer in the same way as above and then mixed with 1 μM ThT or Zn-PPIX. After incubation for 30 min, 2 μM cage **1** was added just before the fluorescence measurement. The fluorescence spectra were collected from 450 to 600 nm with the excitation wavelength of 425 nm for ThT and from 550 to 700 nm with the excitation wavelength of 415 nm for Zn-PPIX. The notion of quenching efficiency (QE) is introduced as below to quantify the binding:

$$QE = \frac{I_0 - I}{I_0}$$

where I is the fluorescence intensity of DNA samples with different concentrations of cage **1** and I_0 is the fluorescence intensity of DNA sample (a mixture of DNA and dye) without cage **1**. The dissociate constant (K_d) in Table S2 was calculated in the same way as shown in our previous work.[1]

S1.4 Circular dichroism measurements

10 μM Tel22 strand was annealed in TSN or TSK (10 mM Tris-H_2SO_4, 10 mM K_2SO_4, pH 7.5) buffer before mixed with different concentrations of cage **1**. CD spectra were collected from 200 to 340 nm for G-quadruplex in 1 mm pathlength cuvettes. The data was acquired on a JASCO J-810 spectropolarimeter (Tokyo, Japan) at room temperature.

S1.5 Native polyacrylamide gel electrophoresis (PAGE)

To compare MT22 and Tel22, the DNA strands were annealed in TSN buffer and then mixed with different concentrations of cage **1** before PAGE analysis. 4 μM DNA with or without cage **1** was mixed with 6 × self-made loading buffer (50% glycerol, 10 mM Tris-H_2SO_4, pH 7.5, self-made loading buffer was used here to avoid the EDTA in the commercial buffer), and then analyzed by 15% native polyacrylamide gel. Low molecular weight DNA ladder was mixed with 6 × commercial loading buffer and added into lane 1. The electrophoresis was conducted in 0.5 × TB buffer (44.5 mM Tris, 44.5 mM boric acid, pH 8.0) with 10 mM Na_2SO_4 at a constant voltage of 110V (10 V/cm) for 1 hour. The gel was immersed in 25% isopropanol for 15 min, then it was stained by 0.01% Stains-All in dark for 4 h. After washing by water and destaining by sun light for 10 min, photo of the gel was taken by a digital camera.

The products of S1 nuclease digestion were analyzed by 15% native polyacrylamide gel in TAE buffer (40 mM Tris, 20 mM acetic acid, 1 mM EDTA, pH 8.0) at the same voltage and stained by GelRed.

S1.6 UV-vis absorbance spectroscopy and dynamic light scattering (DLS) analysis

The samples of 200 μM cage **1** without and with 100 μM Tel22 in TSN buffer were centrifuged at room temperature for 5 min at 10000 rcf. The photos of the tubes in Figure 2e were taken before and after the centrifugation. The solution before centrifugation and supernater after centrifugation were diluted 10 times with the TSN buffer for absorption measurement. In a 10 mm pathlength cuvette, the absorption spectra were collected from 700 to 415 nm on a Cary 300 Bio UV-vis spectrophotometer.

The 100 μM cage **1** alone and mixture of Tel22-cage **1** (ratio 1:2, 50 μM for Tel22) were observed by an optical microscope. Their photos were taken under 40x objective lens.

To prepare the samples for DLS measurement, different concentrations of Tel22 were mixed with cage **1** at the 1:2 molar ratio in TSN buffer at room temperature. The other G4 sequences (5 μM) studied in Fig. 3 were mixed with 10 μM **1** separately in TSK buffer at room temperature. 60 μL of each sample was scanned at 25 °C on a Malvern Zetasizer Nano ZSP. The data shown in Fig. 2a

and Fig. 3b are averages of 10 repeated measurements.

S1.7 S1 nuclease cleavage

8 μM Tel22FQ strand without or with 40 μM cage **1** was mixed with 3U of S1 nuclease in digestion buffer (10 mM Tris-H_2SO_4, 1 mM $ZnSO_4$, 20 mM Na_2SO_4, pH 7.5). The 5 μL of reaction mixture was incubated at 37 °C for 5 min, and then 4 μL of stop buffer (10 mM Tris-H_2SO_4, 70% formamide, 57 mM EDTA, pH 7.5) was added to stop the digestion. It was kept at -20 °C for 10 min before treated by 1 μL of formamide and heated to 95 °C for 3 min. The product was mixed with 2 μL of 6 × loading buffer and then directly analyzed by 15%PAGE. It was diluted 40 times (0.1 μM for Tel22FQ) by TSN buffer before the fluorescence measurement.

S1.8 Molecular docking study

The Tel22 sequence is confirmed to form basket-type G4 structure in Na^+ buffer solution.[2] Thus, antiparallel basket structure (PDB ID 143D) was taken as the binding targets for cage **1**. The crystal structure of **1** was taken from the Cambridge Crystallographic Data Centre (CCDC) with the entry 838413. Molecular docking between the cage and the G4 was performed with software AutoDock4.2.[3] In order to investigate where the cage prefers to bind on the surface of the G4, the grid box was set to be big enough to cover the whole surface of G4 since the binding sites are not known in advance. The grid number was set as 100*100*100 with a grid spacing of 0.375 Å. The center of the grid box was superimposed with the geometric center of the G4. Fully informed particle swarm (FIPS) algorithm[4] was used to optimize the ligand pose by minimizing the binding energy. 500000 moves were evaluated for each docking run so that the searching is converged to an energy minimum.[5] 1000 independent docking runs were conducted between the cage and G4. With the coordinates of 1000 docked complex conformations, the contacts between the atoms of the cage and G4 can be extracted. A contact was defined when the distance between the atoms from G4 and the cage is below a cutoff distance (= 5.0 Å). Contact probability of each atom interacting with the cage was computed for the G4. In this way, the preferred sites of the cage binding can be located on the surface of G4. As shown in Fig. S13, it can be seen that the cage specifically preferred particular sites of the G4 structure. The results showed that the cage may prefer side-binding and the nucleic acids most directly interacting with **1** are A7, G8, A19, G20, G21 and G22. The affinity of optimal binding complex is -11.00 kcal/mol, which suggests that the cage has strong interactions with the G4 structure.

S2 Figures

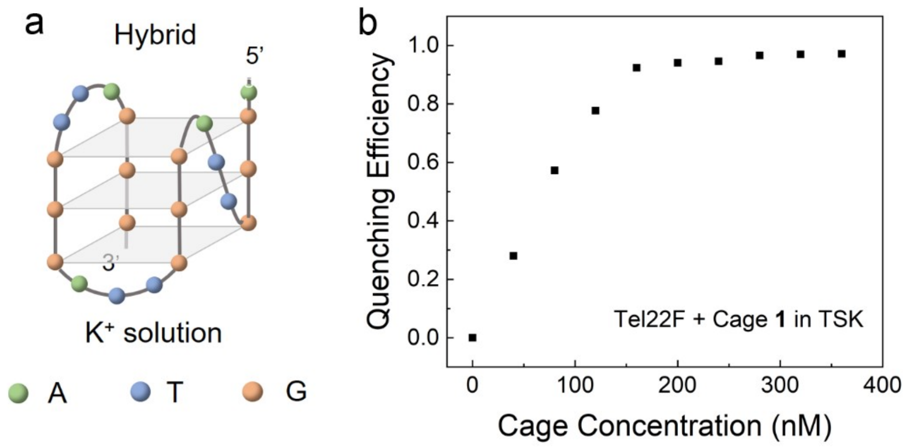

Fig. S1 Fluorescence spectroscopic analysis of the interaction between G4 and cage **1** in TSK buffer (10 mM Tris-H_2SO_4, pH 7.5 with 10 mM K_2SO_4). (a) Structures of G4 in K^+ solutions formed by Tel22F.[6] (b) Dependence of the fluorescence quenching efficiency (QE) for Tel22F (100 nM) at 520 nm on the concentrations of cage **1**.

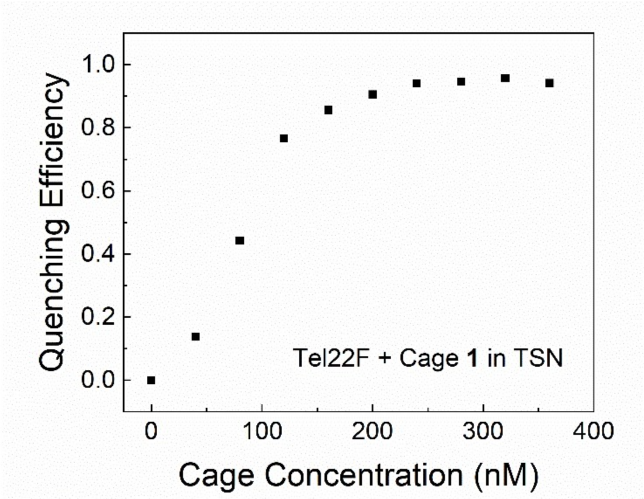

Fig. S2 Dependence of the fluorescence quenching efficiency for Tel22F (100 nM) at 520 nm on the concentrations of cage **1** in TSN buffer.

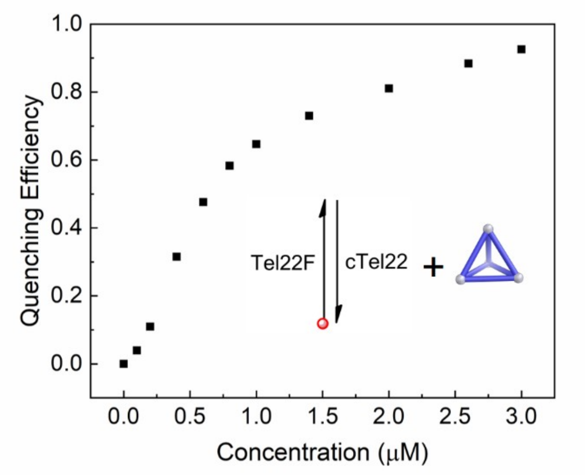

Fig. S3 Dependence of the fluorescence QE for 100 nM duplex Tel22F/cTel22 on the cage **1** concentration in TSM buffer (10 mM Tris-H_2SO_4, pH 7.5 with 10 mM $MgSO_4$). Excitation and emission wavelengths are 495 and 520 nm, respectively.

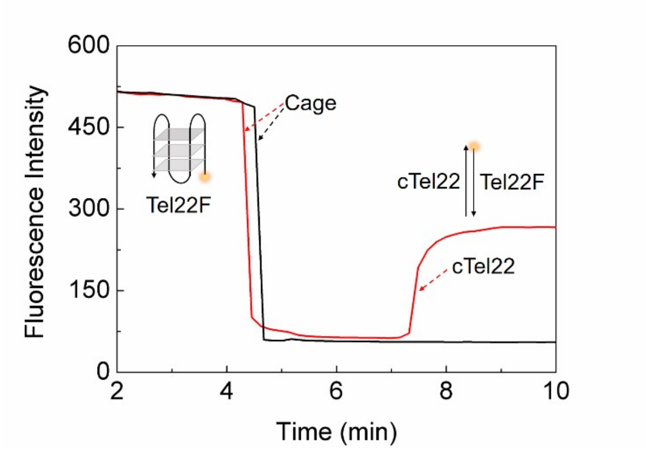

Fig. S4 Dynamic control the fluorescence of 40 nM Tel22F by 120 nM cage 1 and 80 nM cTel22 in TSN buffer. Excitation and emission wavelengths are 495 and 520 nm, respectively. 120 nM cage **1** was added into both samples around 4.5 min to quench the dye, and 80 nM cTel22 was added around 7.5 min to hybridize with Tel22F and release the bound cage.

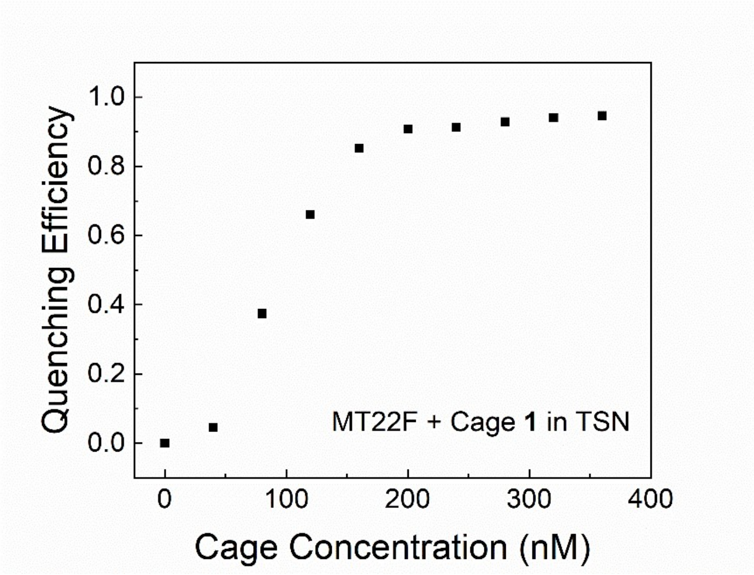

Fig. S5 Dependence of the fluorescence quenching efficiency (QE) for MT22F (100 nM) in TSN buffer at 520 nm on the concentrations of cage **1**.

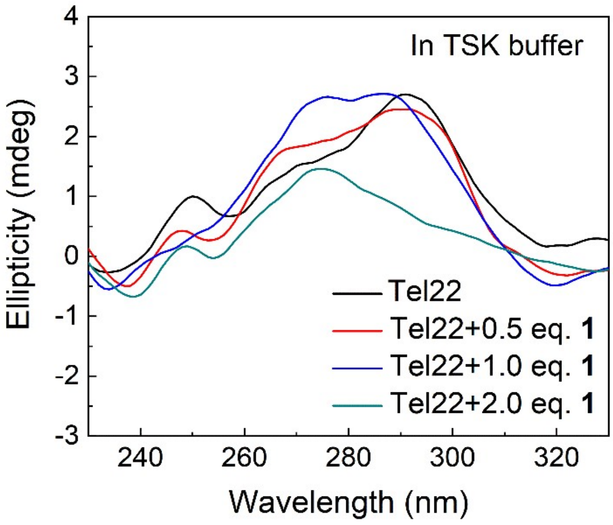

Fig. S6 CD spectra of 10 μM Tel22 in TSK buffer with different concentrations of **1**.

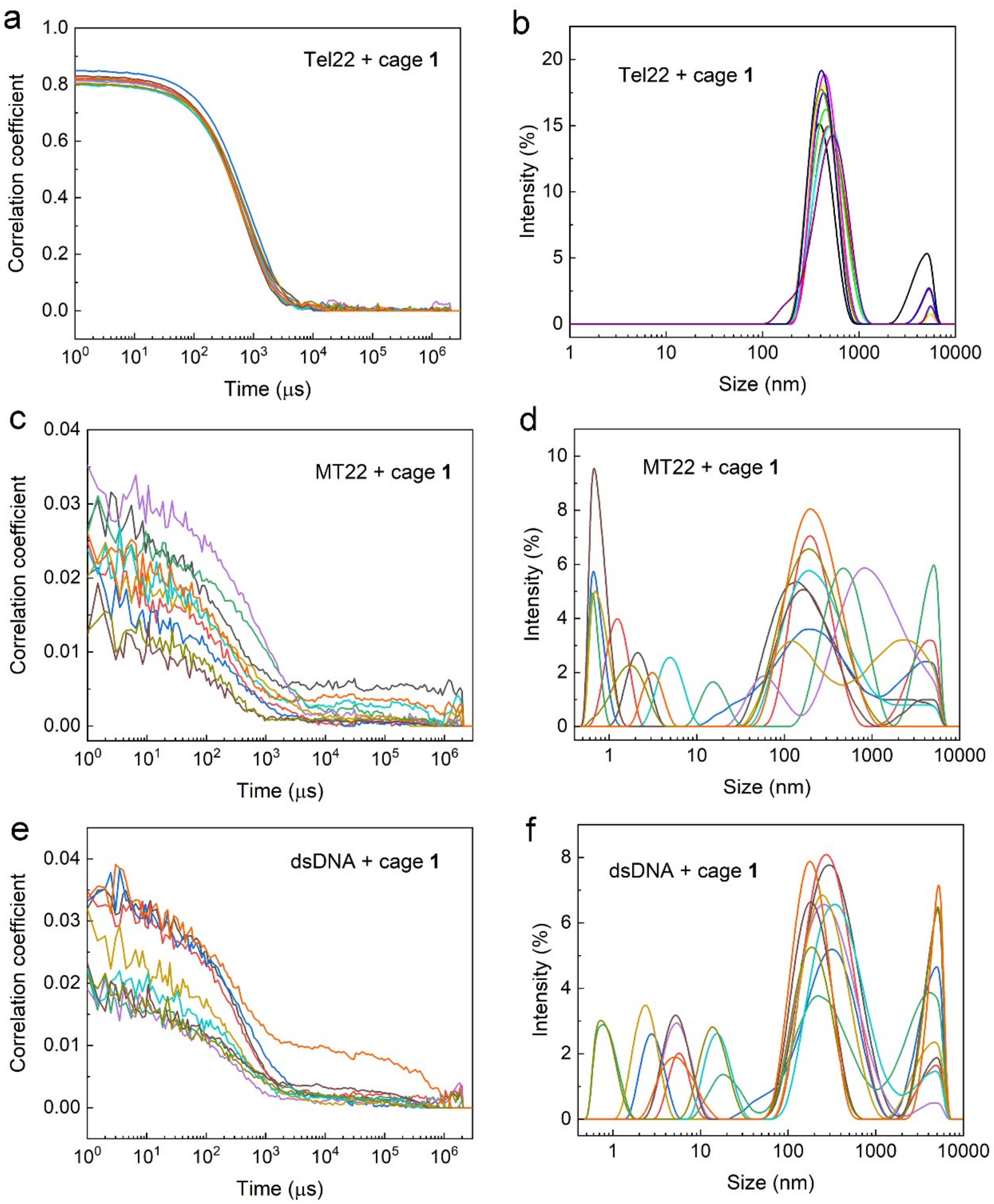

Fig. S7 DLS correlation function curves (a, c, e) and intensity distributions (b, d, f) of DNA-cage **1** mixtures. 1 μM (a, b) Tel22, (c, d) MT22 or (e, f) dsDNA (Tel22·cTel22) was mixed with 2 μM **1** in TSN buffer. Each sample was scanned 10 times, with the data from each measurement shown in different colours. The DLS data for the Tel22-**1** mixture generally overlapped, in contrast to the more scattered intensity distributions of (d) MT22-**1** and (f) dsDNA-**1** mixtures. The correlation function intercepts of MT22-**1** and dsDNA-**1** are both less than 0.1, which indicates the results are complex and not suitable for straightforward DLS analysis.[7]

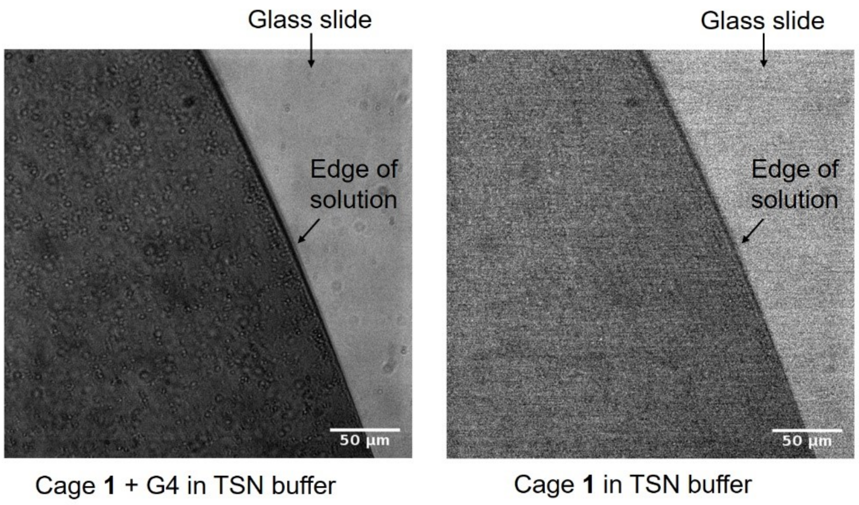

Fig. S8 Photos of 100 μM cage **1** with (left) or without (right) 50 μM Tel22 in TSN buffer taken from an optical microscope. The solution of cage **1** on the right is homogeneous and transparent, but the mixture of cage **1** and Tel22 on the left gets turbid and microspheres with micron size can be observed.

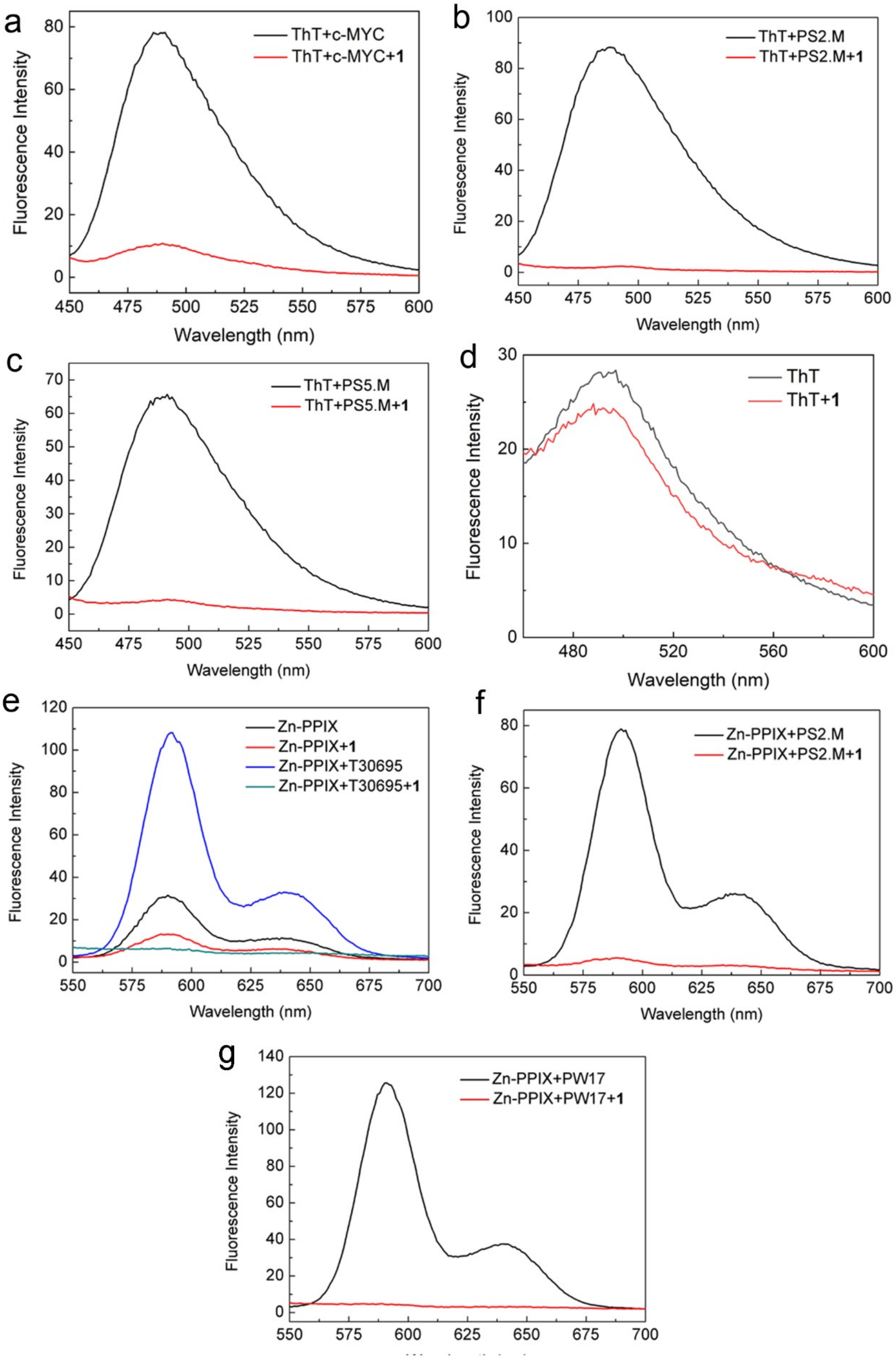

Fig. S9 Cage **1** based quenching of ThT bound on (a) c-myc, (b) PS2.M and (c) PS5.M, and Zn-PPIX bound on (e) T30695, (f) PS2.M and (g) PW17. Sequences of the G-quadruplexes are listed in Table S1. Concentrations for ThT, Zn-PPIX, DNA and cage **1** are 1 μM, 1 μM, 1 μM and 2 μM, respectively. TSK buffer was used for all measurements. ThT and Zn-PPIX were excited at 425 and

415 nm, respectively. The effects of 20 μM cage **1** on the fluorescence of 10 μM ThT were investigated in (d). A wide fluorometer slit width (20 nm) for excitation and emission was used in (d) to enhance the signal.

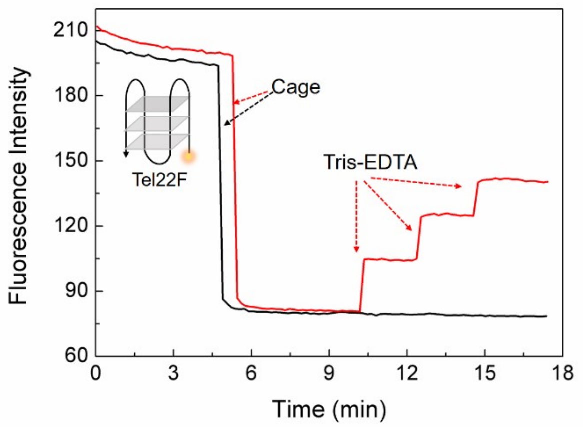

Fig. S10 Recovery of fluorescence by EDTA. 1 mL of 10 nM Tel22F in TSN buffer was excited at 495 nm and fluorescence intensity was recorded at 520 nm. 1 μL of 10 μM cage **1** was added into both samples after around 5 min to quench the dye, and 10 μL of 100 × TE buffer (1 M Tris-HCl, 100 mM EDTA, pH 8.0) was added each time from 10 to 15 min to introduce EDTA into the solution to coordinate Fe^{II} and disassemble the cage.

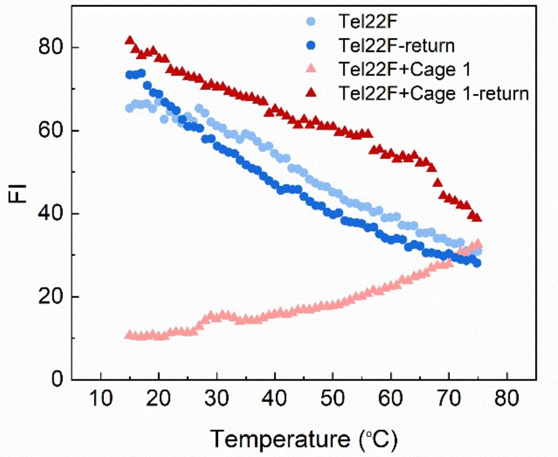

Fig. S11 Effect of heating on the interaction between Tel22F and cage **1**. 20 nM Tel22F without (circle) or with (triangle) 100 nM cage **1** in TSN buffer were heated up from 15°C to 75°C at a rate of 1°C/min and cooled down to 15°C (return) at the same rate. Samples were excited at 495 nm and the fluorescence intensities were recorded at 520 nm. The fluorescence of Tel22F in the mixture was quenched by **1** at the beginning at 15°C. Cage **1** was decomposed with the increased temperature, so the fluorescence was recovered in the return curve. In the sample Tel22F alone, the decrease of the fluorescence intensity with increasing temperature may be caused by the free guanines generated from the unfolded G4 structure

upon heating.

DNA	MT22				
S1	−	+	+	+	+
Cage **1**	−	−	1×	2×	5×

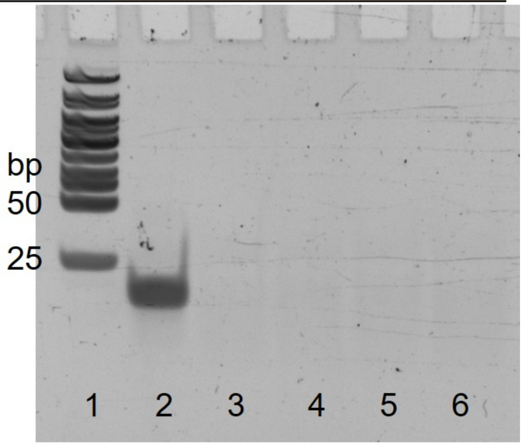

Fig. S12 S1 digestion of MT22 analyzed by 15% PAGE in 0.5 × TB buffer (44.5 mM Tris, 44.5 mM boric acid, pH 8.0). In all cases, **1** was mixed with the DNA before digestion. The content of the mixture loaded into each lane is indicated in the table above the gel. A DNA ladder was added into the first lane.

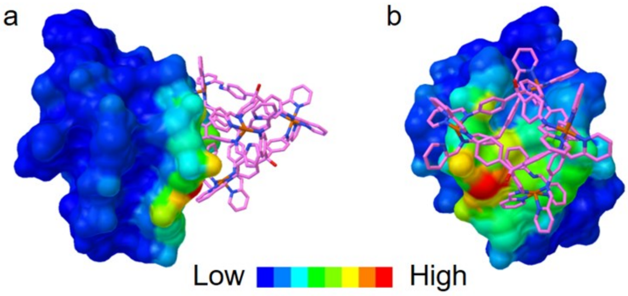

Fig. S13 Binding modes of the cage with basket G4 structure form by Tel22 sequence from different angle of view. The structure of G4 shown in Molecular Surface (MS) style: the contact probabilities are mapped on the surface of G4 and the probability values are represented with color spacing indicating the preference of binding. The cage is shown with sticks. The binding structure in (b) is taken from a viewpoint with a rotation of 90 degrees counterclockwise to (a).

S4 Tables

Table S1.[a] Sequences of DNA strands used in this work.

Strand name	Sequence
Tel22	AGGGTTAGGGTTAGGGTTAGGG
Tel22F	FAM- AGGGTTAGGGTTAGGGTTAGGG
Tel22FQ	FAM- AGGGTTAGGGTTAGGGTTAGGG -Dabcyl
cTel22	CCCTAACCCTAACCCTAACCCT
TA7[b]	AGGGTT*Ap*GGGTTAGGGTTAGGG
TA13[b]	AGGGTTAGGGTT*Ap*GGGTTAGGG
MT22	AGATTTAGTGTTAGAGTTAGAG
MT22F	FAM- AGATTTAGTGTTAGAGTTAGAG
T30695	GGGTGGGTGGGTGGGT
c-myc	TGAGGGTGGGTAGGGTGGGTAA
PS5.M	GTGGGTCATTGTGGGTGGGTGTGG
PS2.M	GTGGGTAGGGCGGGTTGG
PW17	GGGTAGGGCGGGTTGGG

[a] All sequences start from the 5' end. [b] Ap in italic represents the fluorescent base 2-Aminopurine.

Table S2. Dissociate constants (K_d) for different DNA structures interacting with cage **1**. Calculation was based on the data in Fig. S1, S2, S3 and S5.

	ds DNA	Tel22 in TSN	Tel22 in TSK	MT22 in TSN
K_d	1.11 uM	144 nM	94.2 nM	201 nM

References:

[1] J. Zhu, C. J. E. Haynes, M. Kieffer, J. L. Greenfield, R. D. Greenhalgh, J. R. Nitschke, U. F. Keyser, *J. Am. Chem. Soc.* **2019**, *141*, 11358-11362.

[2] (a) J. Dai, M. Carver, C. Punchihewa, R. A. Jones, D. Yang, *Nucleic Acids Res.* **2007**, *35*, 4927-4940; (b) Y. Wang, D. J. Patel, *Structure* **1993**, *1*, 263-282.

[3] G. M. Morris, R. Huey, W. Lindstrom, M. F. Sanner, R. K. Belew, D. S. Goodsell, A. J. Olson, *J. Comput. Chem.* **2009**, *30*, 2785-2791.

[4] Y. Liu, L. Zhao, W. Li, D. Zhao, M. Song, Y. Yang, *J. Comput. Chem.* **2013**, *34*, 67-75.

[5] L. Guo, Z. Yan, X. Zheng, L. Hu, Y. Yang, J. Wang, *J. Mol. Model.* **2014**, *20*, 2251.

[6] A. Ambrus, D. Chen, J. Dai, T. Bialis, R. A. Jones, D. Yang, *Nucleic Acids Res.* **2006**, *34*, 2723-2735.

[7] https://www.materials-talks.com/blog/2020/03/10/correlation-intercept-what-is-it-and-what-does-it-mean/
